# Endoplasmic Reticulum Homeostasis Regulates TLR4 Expression and Signaling in Mast Cells

**DOI:** 10.3390/ijms231911826

**Published:** 2022-10-05

**Authors:** Shatha Boukeileh, Odai Darawshi, Miriam Shmuel, Mohamed Mahameed, Thomas Wilhelm, Priya Dipta, Francesca Forno, Bellam Praveen, Michael Huber, Francesca Levi-Schaffer, Boaz Tirosh

**Affiliations:** 1 The School of Pharmacy, The Hebrew University of Jerusalem, P.O. Box 12065, Jerusalem 9112002, Israel; 2 Institute of Biochemistry and Molecular Immunology, Medical School, RWTH Aachen University, D-52074 Aachen, Germany; 3 Department of Biochemistry, Case Western Reserve University, Cleveland, OH 44106, USA

**Keywords:** ER stress, UPR, ER-phagy, TLR4, asthma, mast cells

## Abstract

The endoplasmic reticulum (ER) is a dynamic organelle that responds to demand in secretory proteins by undergoing expansion. The mechanisms that control the homeostasis of ER size and function involve the activation of the unfolded protein response (UPR). The UPR plays a role in various effector functions of immune cells. Mast cells (MCs) are highly granular tissue-resident cells and key drivers of allergic inflammation. Their diverse secretory functions in response to activation through the high-affinity receptor for IgE (FcεRI) suggest a role for the UPR in their function. Using human cord blood-derived MCs, we found that FcεRI triggering elevated the expression level and induced activation of the UPR transducers IRE1α and PERK, accompanied by expansion of the ER. In mouse bone marrow-derived MCs and peritoneal MCs, the ER underwent a more moderate expansion, and the UPR was not induced following MC activation. The deletion of IRE1α in mouse MCs did not affect proliferation, survival, degranulation, or cytokine stimulation following FcεRI triggering, but it did diminish the surface expression of TLR4 and the consequent response to LPS. A similar phenotype was observed in human MCs using an IRE1α inhibitor. Our data indicate that the ER of MCs, primarily of humans, undergoes a rapid remodeling in response to activation that promotes responses to TLR4. We suggest that IRE1α inhibition can be a strategy for inhibiting the hyperactivation of MCs by LPS over the course of allergic responses.

## 1. Introduction

Secreted proteins fold and mature in the endoplasmic reticulum (ER). In demanding states, where the cell acquires increased secretory capabilities, the protein load in the ER increases and must be met to avoid protein aggregation and cell death. Adaptation to the increased load, referred to as ER stress, evokes mechanisms that monitor the levels of unfolded proteins and prevents their accumulation for risks of aggregation and cell death [1,2].

The main mechanism of adaptation to ER stress in eukaryotic cells is the unfolded protein response (UPR), a collection of signaling pathways aiming at resolving ER stress [3]. In mammalian cells, the UPR comprises three major branches: inositol requiring enzyme 1 α (IRE1α); double-stranded RNA-activated protein kinase (PKR)-like ER kinase (PERK), and activating transcription factor 6 (ATF6), each termed after the ER-transmembrane sensors that gauge the levels of misfolded proteins in the ER lumen and consequently activate their respective downstream signaling cascades.

Studies performed primarily in animal models defective in components of the UPR have provided compelling evidence for prominent physiological roles of this pathway and its dysregulation in diseases. In the immune system, the IRE1α pathway of the UPR regulates multiple effector functions. By virtue of animals deficient in IRE1α in specific cell types, a requirement of IRE1α was demonstrated for B, T, natural killer (NK), and dendritic cells’ (DC) functions [4,5].

IRE1α has both serine threonine kinase and RNA nuclease activities. This unique function of IRE1α allows it to signal via multiple mechanisms. In response to ER stress, IRE1α undergoes auto-phosphorylation, which activates its nuclease activity towards the mRNA of XBP1. This generates the spliced XBP1 (sXBP1) protein, a potent transcription factor that modulates multiple functions in the secretory pathway [6]. In addition to XBP1 mRNA splicing, when stress becomes exaggerated, IRE1α degrades ER-targeted mRNA that contains the recognition hairpin structure, a mechanism referred to as regulated IRE1-dependent decay (RIDD) [7]. In immune cells such as DCs, IRE1α is constitutively activated [8,9]. In other cell types, IRE1α responds to inflammatory signaling, primarily to pattern recognition receptors such as toll-like receptors (TLRs) [10].

Mast cells (MCs) are hematopoietic cells that develop from committed progenitors which differentiate in the bone marrow (BM) and migrate to the peripheral blood. MCs undergo their final maturation in the tissues and are found in virtually all vascularized organs. Their anatomical location in close proximity to external surfaces such as in mucosal tissues, lymphatic vessels, and the skin allows them to respond to external stimuli by releasing a spectrum of mediators including, amongst others, histamine, proteases, cytokines, chemokines, and arachidonic acid metabolites. While MCs are important for host defense by mediating inflammatory responses, their over-activation is also associated with allergic inflammatory and other types of damaging and often chronic reactions, thus being at the limelight of anti-allergy therapy [11]. MC activation is controlled by multiple receptors. The major receptor involved in allergen signaling is FcεRI, which is constitutively expressed on MCs. The cross-linking of IgE-bound FcɛRI by multivalent allergens promotes cell activation and the immediate release of preformed and pro-inflammatory mediators produced de novo [12,13]. The strategic anatomical location of MCs implicates a role in defense against parasites and bacteria by stimulation of TLRs. Accordingly, allergic symptoms are often increased in the presence of pathogens at the site of allergen exposure [14] that have a facilitated invasion because of the loss of epithelial integrity in allergic diseases. Therefore, synergistic interactions between FcɛRI and TLRs may have relevance to the exacerbation of IgE-mediated allergic diseases by infectious agents, as in the case of viral and bacterial infections that have been associated with asthma exacerbations [15]. This effect was observed for both human and murine MCs upon prolonged exposure to TLR agonists, specifically TLR4 ligands (lipopolysaccharide (LPS)), in combination with IgE receptor cross-linking. The TLR4/FcεRI co-activation enhances degranulation, the secretion of leukotrienes, and the induction of IL-6, MCP-1, and MIP-1α production [16].

The UPR pathways, primarily the IRE1/XBP1 arm, play seminal roles in immune cell activation, including B cells [17], T cells [18], NK cells [19], dendritic cells [8], and eosinophils [20]. Despite their developed secretory activities, little is known about the ER stress in MCs. Nam et al. showed that preincubation of MCs with the IRE1α inhibitor 4μ8C inhibits MC degranulation and the induction of the pro-inflammatory cytokine TNF; a detailed analysis of UPR activation was not provided [21]. We have previously reported a constitutive activation of the UPR in human MC leukemia (MCL) cells. In these cells, the inhibition of IRE1α nuclease activity compromised proliferation and survival [22]. However, a role for IRE1α in primary human MCs was not reported. Here we investigated the role of IRE1α in primary MC activation, both human and mouse. We found that IRE1α expression and activation is rapidly induced by FcεRI activation in human MCs as part of a general expansion of the ER. While mouse MCs similarly induce IRE1α by FcεRI activation, the activation of IRE1α was not observed. The deletion of IRE1α resulted in a reduced response to LPS in correlation with a reduced surface expression of TLR4. We propose that FcεRI, in a mechanism that involves IRE1α activation, promotes the expansion of the ER. This supports the cellular pool of TLR4 and its signaling. Hence, inhibitors of IRE1α may be useful in curtailing the synergism between FcεRI and TLR4.

## 2. Results

### 2.1. Activation of Human Cord Blood-Derived Mast Cells (CBMCs) Promotes the Expression of IRE1α and PERK

**Figure 1:**
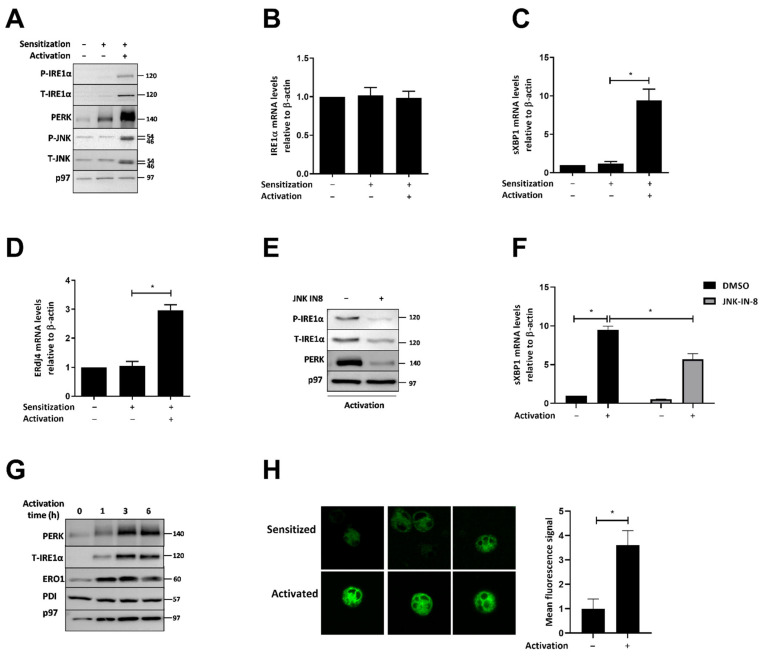
Activation of CBMCs promotes expression of IRE1α and PERK. CBMCs were sensitized by culturing them with human myeloma IgE (0.3 μg/mL). Three days later, cells were activated by rabbit anti-human IgE Ab (5 μg/mL, 4 h). (**A**) Equal amounts of whole cell extracts were analyzed by immunoblotting for phosphorylated and total IRE1α, PERK, phosphorylated and total JNK, and p97 as a loading control. A representative image of three independent experiments is shown. RT-qPCR analyses of (**B**) IRE1α, (**C**) sXBP1, and (**D**) ERdj4 following the indicated treatments, represented as the mean of three independent experiments normalized to β-actin ± SD. * *p* < 0.05. (**E**) IgE-sensitized CBMCs were pretreated with 3 µM JNK-IN-8 for 1 h, followed by activation with rabbit anti-human IgE Ab (5 μg/mL, 4 h). Equal amounts of whole cell extracts were analyzed by immunoblotting for phosphorylated and total IRE1α, PERK, and p97 as a loading control. (**F**) RT-qPCR analysis of sXBP1 following the indicated treatments, represented as the mean of relative mRNA levels normalized to β-actin ± SD, * *p* < 0.05. (**G**) CBMCs were sensitized with human myeloma IgE (0.3 μg/mL, 3 days), followed by activation with rabbit anti-human IgE Ab (5 μg/mL) for 1, 3, and 6 h. Equal amounts of whole cell extracts were analyzed by immunoblotting for PERK, total IRE1, ERO1, PDI, and p97 as a loading control. A representative image of three independent experiments is shown. (**H**) Fluorescence of ER-Tracker (green marker) in CBMCs following IgE-mediated activation for 4 h, analyzed by confocal microscopy (60× with immersion oil). The fluorescence signal from 50 cells was quantified. Data are expressed as mean intensity ±SD. * *p* < 0.05.

To measure the activation of the UPR in primary human MCs, we generated CBMCs. Following activation by FcεRI cross-linking, we observed a strong induction in IRE1α expression from levels below detection limits to an overt expression. This was accompanied by the detection of IRE1α phosphorylation. A similar expression pattern was observed for PERK with an indication of activation. The c-Jun N-terminal kinases (JNK) is activated downstream to FcεRI [23]. Total levels of JNK and its phosphorylation were also increased similarly to the UPR transducers IRE1α and PERK (Figure 1A). The analysis of IRE1α mRNA did not show a significant difference, suggesting that the increase in IRE1α is post-transcriptional (Figure 1B). Consistent with the phosphorylation of IRE1α, mRNA levels of sXBP1 were induced approximately 10-fold (Figure 1C). mRNA levels of ERdj4, a direct target of sXBP1 [24], were also increased (Figure 1D). These data show that human MCs respond to IgE-mediated activation by inducing the UPR. Our previous work showed that, in response to ER stress, JNK is induced in the human mast cell line HMC1.2 [22]. The induction in both total and phosphorylated JNK in human primary MCs following activation has been reported [25]. We reasoned that the two are interconnected. The inclusion of the JNK inhibitor, JNK-IN-8, together with FcεRI activation inhibited the phosphorylation of IRE1α, the induction of both IRE1α and PERK (Figure 1E), and the downstream activation of XBP1 splicing (Figure 1F).

The fact that both IRE1α and PERK were induced by MC activation suggested that this may be related to more general processes of the ER, rather than to specific regulation. One such process is the expansion of the ER, which commonly occurs in immune cells once differentiating into effectors [18,26]. We performed a time course analysis for ER proteins expression. PERK and IRE1α levels were already higher 1 h after MC activation. This induction reached a steady state at 3 h. The expression pattern of the ER-resident protein ERO1 was similar to that of PERK and IRE1α, suggesting that this regulation is not unique to the UPR transducers. In contrast, we did not observe a change in the expression of the soluble ER protein PDI (Figure 1G). We conclude that, following activation, the ER of MCs undergoes a remodeling that includes the induction of a subset of its proteins. To visualize the ER, we used the dye ER-Tracker Green, which binds ATP-sensitive K+ channels that are prominent on the ER. Analysis of different optic fields showed that the IgE-mediated activation of CBMCs increases staining by more than threefold (Figure 1H). While this increase in staining may be a consequence of multiple factors, it supports a role for rapid ER expansion in MC activation downstream to FcεRI.

### 2.2. Inhibition of IRE1α Nuclease Activity Decreases the Secretory Response to IgE-Mediated Activation in CBMCs

**Figure 2:**
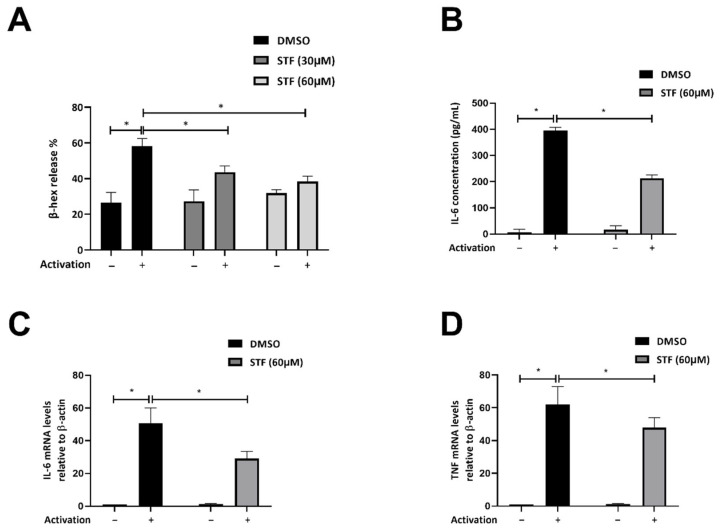
Inhibition of IRE1 nuclease activity decreases the secretory response to IgE-mediated CBMC activation. Sensitized CBMCs were pretreated with DMSO or STF (30 or 60 μM) 1 h prior to activation with rabbit anti-human IgE Ab (5 μg/mL). Thirty minutes after activation, (**A**) supernatants and cell pellets underwent chromogenic assay for β-hex quantification. Data are shown as mean ± SD. * *p* < 0.05. (**B**) Supernatants were collected following 4 h of activation and analyzed for IL-6 by ELISA. Each bar represents the average of three independent measurements ± SD. *p < 0.05. (**C**) RT-qPCR analysis of IL-6, and (**D**) TNF following 4 h of activation. The average of mRNA levels normalized to β-actin ±SD of three independent experiments is shown. * *p* < 0.05.

We aimed to determine whether IRE1α affects MC functionality. CBMCs were therefore sensitized with human myeloma IgE and activated in vitro with polyclonal rabbit anti-human IgE for 30 min in the presence and absence of STF-083010 (STF), a protected aldehyde compound that forms a Schiff base with catalytic lysines of the IRE1α nuclease site. This compound blocks both RIDD and XBP1 mRNA splicing. IRE1α inhibition in CBMCs showed a dose-dependent reduction in β-hexosaminidase (β-hex) release (Figure 2A). This is consistent with previous data [27], but could be a result of off-target activities of the drug. Proinflammatory cytokines mRNA levels and release, represented by IL-6 and TNF, were also diminished in CBMCs pretreated with STF (Figure 2B–D). We conclude that IRE1α nuclease activity is required for the maximal stimulation of human MCs.

### 2.3. Peritoneal Murine Mast Cell Activation through FcεRI Promotes Expression of IRE1α without UPR Activation

**Figure 3:**
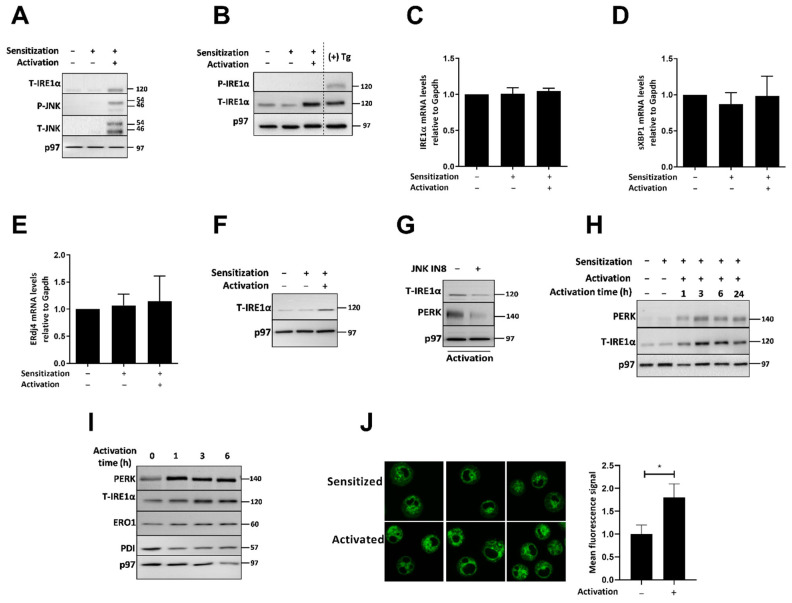
PMC activation through FcεRI promotes expression of IRE1α without UPR activation. (**A**) WT PMCs were sensitized with mouse IgE-anti-DNP (0.5 µg/mL, 18 h) and then activated with DNP-BSA (50 ng/mL, 4 h). Equal amounts of whole cell extracts were analyzed by immunoblotting for total IRE1α, phosphorylated and total JNK, and p97 as a loading control. A typical immunoblot is shown. (**B**) WT PMCs were treated for 4 h as mentioned in (**A**) or treated with Tg (1 µg/mL) as a positive control. Equal whole cellular protein extracts were analyzed by immunoblotting for phosphorylated and total IRE1α, and p97 as loading control. Shown is a representative image of three independent experiments. (**C**) RT-qPCR analyses of IRE1α, (**D**) sXBP1, and (**E**) ERdj4 following 4 h of activation represented as the average of the mRNA levels normalized to GAPDH ± SD. (**F**) PMCs were sensitized with rat myeloma IgE (1 µg/mL, 18 h) followed by activation with goat anti-rat IgG F(ab’)_2_ Ab (1 µg/mL, 4 h). Identical quantity of total cellular protein extracts was analyzed by immunoblotting against total IRE1α and p97 as a loading control. (**G**) IgE sensitized WT PMCs with IgE-anti-DNP (0.5 µg/mL, 18 h) were pretreated with 3 µM JNK-IN-8 for 1 h followed by activation with DNP-BSA (50 ng/mL, 4 h). An identical quantity of total cellular protein extracts was analyzed by immunoblotting against total IRE1α, PERK, and p97 as a loading control. (**H**) WT PMCs were sensitized with mouse IgE–anti-DNP (0.5 µg/mL, 18 h), followed by activation with DNP-BSA (50 ng/mL) for 1, 3, 6, or 24 h. An identical quantity of protein was analyzed by immunoblotting against PERK, total IRE1α, and p97 as a loading control. (**I**) WT PMCs were sensitized with mouse IgE–anti-DNP (0.5 µg/mL, 18 h), followed by activation with DNP-BSA (50 ng/mL) for 1, 3, and 6 h. Equal amounts of whole cell extracts were analyzed by immunoblotting for PERK, total IRE1α, ERO1, PDI, and p97 as a loading control. (**J**) Fluorescence of ER-Tracker in PMCs following IgE-mediated activation for 4 h, analyzed by confocal microscopy at magnification of 60× with immersion oil. Mean fluorescence signal from 50 cells was quantified using ImageJ 1.53t software (U. S. National Institutes of Health, Bethesda, MD, USA). The average intensity and standard deviation are shown. * *p* < 0.05.

To examine the role of the UPR in murine MC function, we isolated peritoneal MCs (PMCs) from WT mice and expanded them ex vivo for three weeks in the presence of SCF and IL-3. Activation of PMCs by FcεRI cross-linking induced IRE1α and JNK expression (Figure 3A). In contrast to human MCs, we did not detect phosphorylated IRE1α (Figure 3B). IRE1α mRNA was not affected (Figure 3C). The absence of IRE1α phosphorylation was supported by the absence of XBP1 splicing (Figure 3D) and no change in the mRNA levels of ERdj4 (Figure 3E). We conclude that IRE1α is induced in primary mouse MCs in the absence of its activation. To exclude that this is related to the reagents used for activation, we used rat myeloma IgE followed by cross-linking with goat anti-rat IgG F(ab’)_2,_ instead of using the DNP-BSA and anti-DNP monoclonal IgE. IRE1α was similarly induced by this activation as well (Figure 3F). A 1 h pretreatment with JNK-IN-8 was sufficient to inhibit IRE1α and PERK induction (Figure 3G), similar to CBMCs. We then aimed to check whether the induction of IRE1α is accompanied by that of PERK. As observed for human MCs, the expression of IRE1α and PERK was induced, and reached a steady state approximately 3 h after activation (Figure 3H). PERK electromobility was not altered, indicating that the increase in expression occurred in the absence of ER stress (Figure 3I). Visualization of the ER by an ER tracker showed a 45% increase in staining upon activation (Figure 3J).

We conducted a similar set of analyses for bone marrow-derived MCs (BMMCs). Similar to PMCs, IRE1α expression was induced in BMMCs in a post-transcriptional mechanism with no evidence of UPR activation (Supplemental Figure S1A–E). This increase was observed with the two sets of activation reagents (Supplemental Figure S1F). The increase in the IRE1α level without evidence of UPR activation suggested that, at the activation state, if ER stress develops, MCs will respond stronger than at the naïve state. To address this, we combined IgE-mediated activation at increasing concentrations with increasing concentrations of thapsigargin (Tg), an inhibitor of SERCA, which activates the UPR. We observed a clear additive response with respect to the absolute levels of phosphorylated IRE1α. A combination of IgE-dependent activation at 50 ng/mL reached the maximal IRE1α phosphorylation in the presence of 100 ng/mL of Tg (Supplemental Figure S1G). We conclude that, in murine MCs, the UPR machinery is induced upon IgE-mediated activation in anticipation of ER stress. Once triggered, it is poised to respond more strongly. This implies that, although murine mast cell activation via FcɛRI does not activate the UPR, the resulting enhanced expression of the IRE1α does sensitize the PMCs to ER stress.

### 2.4. The Absence of IRE1α Does Not Affect PMCs’ Secretory Response to IgE-Mediated Activation

Our data show that, while human MCs respond to activation by generating ER stress, murine MCs do not. However, for both species, expansion of the ER occurs in a manner that elevates the cellular expression of the UPR transducers. To further investigate the role of the IRE1/XBP1 pathway in MC function, we generated XBP1 and IRE1α/XBP1 MC-specific knockout mice. We used the transgenic Mcpt5-Cre mice to induce the recombination of IRE1α and/or XBP1 alleles. We deliberately distinguished between XBP1 KO and IRE1α KO, owing to the gain-of-function of IRE1α in the absence of XBP1, which results in an elevation of the RIDD activity of IRE1α, as we and others have shown [28,29]. When the mouse lines were established, we noticed a partial deletion of the targeted allele. We therefore crossed the mice to a YFP reporter for a direct assessment of the efficiency of recombination and for separating the KO from the non-recombined cells.

We assessed XBP1 recombination by a PCR analysis for the recombined locus. The efficiency of IRE1α knockout was assessed by immunoblotting, since IRE1α recombination generates a truncated protein that lacks the nuclease domain. Analysis of XBP1 locus in PMCs showed a complete recombination. As expected, a higher expression of IRE1α was observed in XBP1 KO PMCs as compared to WT PMCs [29]. A partial KO was observed for IRE1α in DKO PMCs (Supplemental Figure S2A). A visualization of the cells did not show a clear morphological and/or phenotypical difference between WT and knockout PMCs upon staining with alcian blue and safranin (Supplemental Figure S2B). Flow cytometry showed that the YFP-positive cells expressed normal levels of the MC surface markers, FcεRI and KIT, indicating complete differentiation (Supplemental Figure S2C). Utilizing our mouse model, we investigated the effect of the UPR on MC survival by analyzing the proliferation of PMCs. Flow cytometry tracking of YFP expression percentages showed that YFP-positive PMCs proliferate and mature normally (Supplemental Figure S2D). Tracking the forward and side scatter, which imply for cell size and granularity, did not show significant differences between WT and XBP1 or IRE1α-deficient cells (Supplemental Figure S2E). Thus, despite the partial deletion of IRE1α in PMCs, we conclude that IRE1α and XBP1 are not required for MC differentiation in mice. This allowed us to set up studies in which the biological effects during various stimulatory settings can be comparatively analyzed.

**Figure 4:**
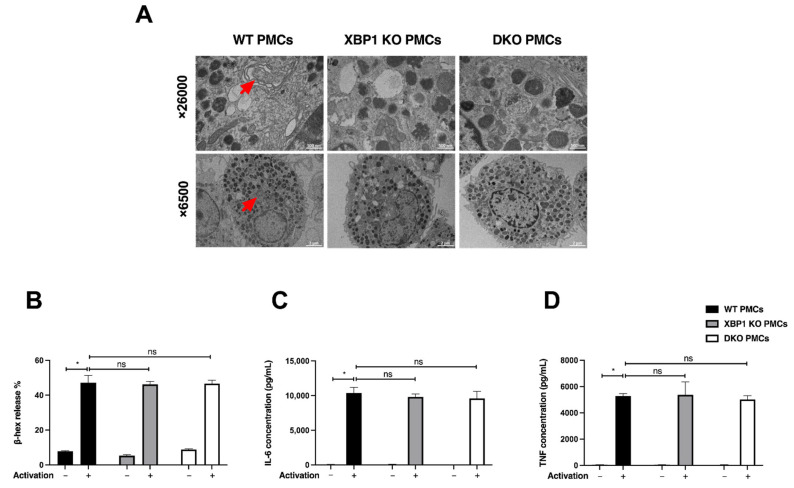
The absence of IRE1α does not affect PMC secretory response to IgE-mediated activation. (**A**) PMCs expanded from the peritoneal lavage of the WT, XBP1 KO, and DKO strain mice were sorted for YFP expression and visualized by TEM. Typical cells from 2 independent separations are shown. Red arrows show the perinuclear ER, which is not discerned in the DKO PMCs. Bars: 500 nm (upper panel) and 2 µm (lower panel). (**B**) PMCs were sensitized with mouse IgE-anti-DNP (0.5 µg/mL, 18 h), followed by activation with DNP-BSA (50 ng/mL, 1 h). Following activation, supernatants and cell pellets underwent a chromogenic assay for β-hex quantification. Data are shown as the mean ± SD of three independent experiments. Supernatants were analyzed for secreted cytokines as a response to IgE-mediated activation of 4 h in YFP-sorted XBP1 KO PMCs and DKO PMCs, as compared to WT PMCs. The amounts of (**C**) IL-6 and (**D**) TNF were determined by ELISA. Each bar represents the average ± SD of three independent experiments. * p < 0.05.

The lack of IRE1α in plasma cells does not affect differentiation but leads to a degenerated ER [30]. In XBP1 KO plasma cells, the ER is distended [29]. We therefore wanted to assess whether IRE1α and XBP1 KO PMCs also show an atrophied ER. We analyzed the YFP-positive PMCs of WT, XBP1 KO, and DKO strains by transmission electron microscopy. Similar to plasma cells, we observed more ER distention in the XBP1 KO cells. The typical perinuclear region in the WT cells that is rich in ER membranes was not observed in the DKO PMCs. We conclude that the IRE1/XBP1 pathway of the UPR is required to develop the ER of MCs (Figure 4A). However, a series of functional tests on the MCs, such as β-hex release and the measurement of pro-inflammatory cytokines (IL-6 and TNF) in the supernatants following IgE-dependent stimulation did not reveal a significant difference (Figure 4B–D). We conclude that, despite affecting the ER, the IRE1/XBP1 pathway is not required for the storage and release of proinflammatory mediators upon the IgE-mediated stimulation of mouse BMMCs.

### 2.5. TLR4 Surface Expression and Function Is Supported by the IRE1/XBP1 Pathway

**Figure 5:**
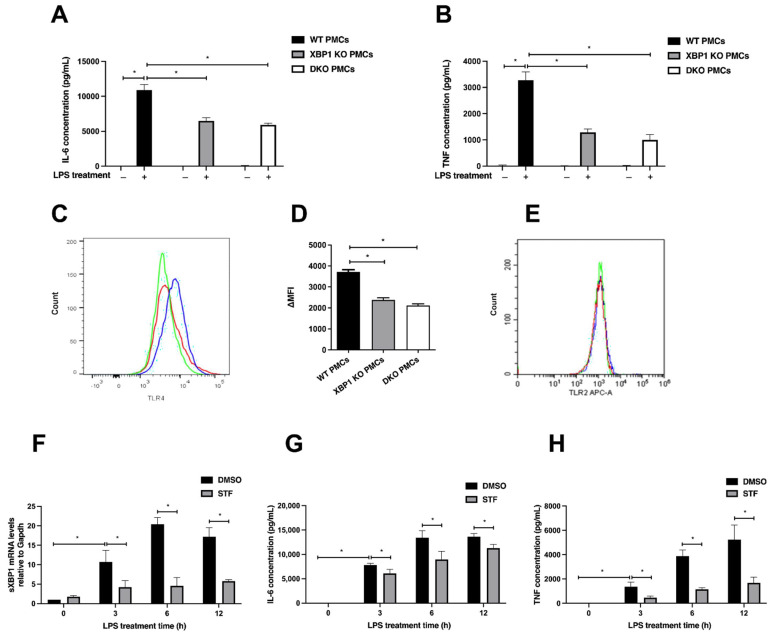
TLR4 surface expression and function is supported by the IRE1/XBP1 pathway. TNF and IL-6 levels in the supernatants in response to LPS stimulation (1 μg/mL, 4 h) were measured by ELISA for an equal number of YFP-sorted XBP1 KO and DKO PMCs, as compared to WT PMCs. The average of three independent experiments ± SD is shown for (**A**) IL-6 and (**B**) TNF. * *p* < 0.05. (**C**) Flow cytometry analysis of TLR4 cell surface expression of XBP1 KO (green histogram) and DKO PMCs (red histogram), as compared to WT PMCs (blue histogram). (**D**) Quantification of TLR4 expression levels in WT, XBP1 KO, and DKO PMCs. Data are presented as the average ± SD of the mean fluorescence intensity minus the background (ΔMFI) of three independent analyses. * *p* < 0.05. (**E**) Flow cytometry analysis of TLR2 expression in WT, XBP1 KO, and DKO PMCs (blue, green, and red histograms, respectively). (**F**) RT-qPCR analysis of sXBP1 in PMCs treated with DMSO or 60 μM STF, 1 h prior to stimulation with LPS (1 μg/mL) for 3, 6, and 12 h. Bars are represented as the mean of the relative mRNA levels normalized to GAPDH ± SD of three independent experiments. Cytokines levels in the supernatants for (**G**) IL-6 and (**H**) TNF were determined by ELISA. Each bar represents the mean ± SD of three independent experiments. * *p* < 0.05.

As being sentinels of the immune system, MCs also recognize pathogens directly via the PRRs expressed on their surface, including toll-like receptors (TLRs). Clinically, asthmatic patients are more susceptible to bacterial infections [31]. Mice knockouts for TLR2, TLR4, or MyD88 develop a less severe response to allergic airway disease [32]. The connection between TLR4 and ER stress has been investigated primarily in myeloid cells, macrophages, and dendritic cells. It was shown that TLR4 triggering by LPS promotes ER stress and activates IRE1α [33]. However, little is known whether IRE1α regulates TLR4 signaling. When examined in PMCs, the deletion of XBP1 alone or in conjunction with IRE1α resulted in a significant reduction in IL-6 (Figure 5A) and TNF (Figure 5B) release following PMC stimulation with LPS. Flow cytometry analysis of surface TLR4 expression on PMCs showed a reduced level in XBP1 KO and DKO PMCs when compared to WT PMCs (Figure 5C,D). This was specific to TLR4, as no change in the expression of TLR2 was detected (Figure 5E). We analyzed the mRNA levels of the TLRs in the different MC genotypes. No significant difference was observed for any of them (Supplemental Figure S3). TLR4 stimulation by LPS resulted in IRE1 activation, manifested by XBP1 mRNA splicing (Figure 5F), which was reduced by the IRE1α inhibitor STF within 3 h after stimulation for both IL-6 (Figure 5G) and TNF (Figure 5H). We conclude that the IRE1/XBP1 pathway of the UPR supports the response of TLR4 in MCs.

### 2.6. IRE1α Activation Supports the Cooperativity between TLR4 and FcεRI in CBMC Activation

**Figure 6:**
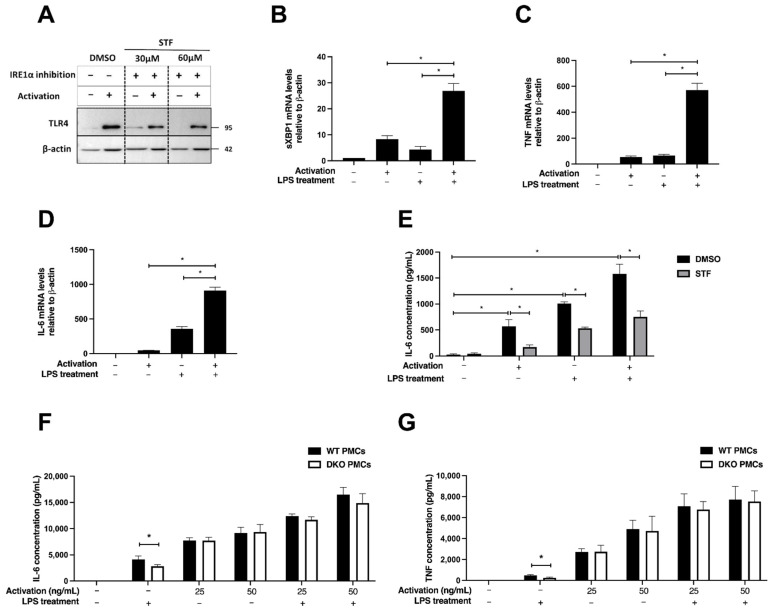
IRE1 activation supports cooperativity between TLR4 and FcεRI in CBMCs. (**A**) IgE-sensitized CBMCs were pretreated with DMSO or STF (30 or 60 μM) for 1 h followed by activation with rabbit anti-human IgE Ab (5 μg/mL, 4 h). Equal amounts of whole cell protein extracts were analyzed by immunoblotting for TLR4 and p97 as a loading control. RT-qPCR analysis of sensitized CBMCs after activation with rabbit anti-human IgE Ab (5 μg/mL), stimulation with 100 ng/mL LPS, or both treatments for 4 h, for (**B**) sXBP1, (**C**) TNF, and (**D**) IL-6. Bars represent the mean of relative mRNA levels normalized to β-actin ± SD of three independent experiments. * *p* < 0.05. (**E**) IgE-sensitized CBMCs were pretreated with DMSO or STF (60 μM) for 1 h followed by activation with rabbit anti-human IgE Ab (5 μg/mL), 100 ng/mL LPS, or both for 4 h. IL-6 levels in the supernatants were quantified by ELISA. Bars represent the mean ± SD of three independent experiments. * *p* < 0.05. PMCs were sensitized overnight with mouse IgE-anti-DNP (0.5 µg/mL, 18 h). Later, cells were stimulated with 100 ng/mL LPS, activated with DNP-BSA (25 or 50 ng/mL), or stimulated with both for 4 h. Levels of IL-6 (**F**) and TNF (**G**) in supernatants were determined by ELISA. Each bar represents the mean ± SD of three independent experiments. * *p* < 0.05.

To examine the role of IRE1α in CBMC activation by LPS and FcεRI, we evaluated TLR4 expression following FcεRI cross-linking in the presence and absence of STF. We did not detect TLR4 at the cell surface by flow cytometry using various monoclonal antibodies. This was reported also by others and is probably related to the high granularity of CBMC, which interferes with fluorescence detection [34]. TLR4 was readily detected by immunoblotting. We observed a strong induction in TLR4 total level of expression following activation. A preincubation with the IRE1α inhibitor STF curtailed the induction (Figure 6A). This suggests that the ER remodeling in human MCs following activation supports a higher level of TLR4 expression. When the effect on IRE1α activation was examined, we observed a marked increase in XBP1 mRNA splicing when LPS was combined with FcεRI cross-linking (Figure 6B). A strong induction in IL-6 and TNF expression was observed when the two stimuli were combined (Figure 6C,D). Inclusion of the IRE1α inhibitor diminished the additive effect of TLR4 and FcεRI (Figure 6E). We conclude that IRE1α supports this cross-talk. Next, we investigated whether IRE1α is needed to support the cross-talk between TLR4 and FcεRI even when the UPR is not activated in the course of IgE-dependent activation, as seen in PMCs. IgE-sensitized PMCs were stimulated with IgE cross-linking using DNP-BSA or with LPS, alone or with the combination for 4 h. The coactivation of FcεRI and TLR4 amplified the release of the proinflammatory responses, which was modestly diminished in the DKO PMCs (Figure 6F,G). We conclude that IRE1α activation, rather than its presence, supports the synergism or additive effect between FcεRI and TLR4 signaling for maximal MC activation.

## 3. Discussion

Cellular organelles communicate with each other physically and functionally. In this respect, little is known on how signals for degranulation affect the early secretory pathway. We argue that MCs are a unique model for addressing these dynamics. Physiologically, MCs are long-lived tissue-resident cells with important roles in many inflammatory settings, including allergic reactions and host defense against pathogens. The cytoplasm of MCs is teeming with granules containing pro-inflammatory mediators such as tryptase, β-hex, and histamine. Triggering the cells via FcεRI causes a massive release of the granules, also named compound exocytosis. The release of the pre-stored mediators is accompanied with the transcription upregulation of multiple pro-inflammatory cytokines and other mediators that utilize the canonical secretory pathway for a rapid secretion. To account for this function, we propose that MCs undergo ER remodeling.

Here, we show a role for IRE1α for ER remodeling after activation. Unexpectedly, we unravel a role for this remodeling in promoting the surface expression of TLR4. We show that human and mouse MC activation is associated with a rapid expansion of the ER after MC activation (Figure 1, Figure 2 and Figure 3). While UPR activation is not necessary for the expansion, as seen for mouse MCs, it provides support and enhancement to ER expansion. As part of the expansion, the expression of the UPR mediators increases without a significant elevation in the mRNA levels of IRE1α. The similar effect on IRE1α and PERK expression suggest that the expansion of the ER may improve the recruitment of certain mRNA to the ER translocons in a manner that promotes the efficiency of their translation (Figure 1). The higher expression of the UPR sensors allows them to respond more strongly to ER stress conditions (Supplemental Figure S1).

We assume that the main trigger for ER expansion is a calcium ion leak from the ER [35]. The importance of calcium for MC degranulation has been known for more than 50 years [36]. The fact that store-operated calcium release-activated calcium channels (CRACs) are required for the degranulation and production of various mediators [37] indicates that the activation of MCs is associated with a transient depletion of ER calcium storage, a well-established mechanism for the induction of ER stress. In addition to calcium, the transcriptional contribution of ER stress for ER expansion is conserved from yeast [38] to mammalian cells [39] and is thus expected to contribute to the phenotype. This activity is mostly ascribed to sXBP1 by promoting lipid biosynthesis and the protein machinery needed to increase the number of ER sheets [40]. A less understood pathway is ER-phagy, which directs portions of the ER to lysosomal degradation. Following stress conditions, including ER stress, several ER-phagy receptors decorate regions of the ER recognized for autophagy [41]. One of these receptors, FAM134B, is transcriptionally regulated by JNK [42]. The engagement of ER-phagy allows for a rapid reorganization of the ER when cells recover from ER stress [43]. Whether ER-phagy, on its various flavors, is engaged in MCs and contributes to the remodeling of the ER in activated MCs has not been addressed here. More generally, since autophagy is required for efficient degranulation of MCs downstream to FcεRI [44], it is plausible that ER-phagy and/or macro-autophagy is constitutive and subjected to inhibition, once MCs are activated. In support of this possibility is the role of ORMDL3 [45]. Mutated ORMDL3 is a risk factor for childhood asthma [46]. The WT allele is a negative regulator of MC activation [47] and, when overexpressed, provokes ER stress [48] and inhibits autophagy [49]. Thus, ER stress and ER-phagy might be interconnected in MCs in a manner that contributes to ER expansion. We suggest that ORMDL3 may regulate these processes upstream to IRE1α.

The specific influence of ER remodeling on TLR4 expression and signaling without affecting its mRNA levels was unexpected. The missing link may be the expression of MD-2, a co-receptor necessary for TLR4 signaling, as it has been reported that JNK regulates MD-2 expression in the promyelocytic leukemia HL-60 cells [50]. TLR4 assembles with its accessory molecule MD-2 in the ER in a poorly understood mechanism that requires gp96 [51,52]. An additional ER protein PRAT4A regulates the ER exit of TLR4 and additional TLRs [53]. When overexpressed, as a GFP fusion, the majority of TLR4 resides in the ER [54]. We suggest that the ER in MCs serves as the cellular reservoir of TLR4 and its steady-state expression level at the cell surface, and its ability to signal is directly proportional to its amount in the ER. When reduced in size, due to the genetic deficiency of IRE1α, the levels of surface TLR4 are reduced (Figure 4 and Figure 5). Other TLRs, such as TLR2, which signals from endosomal compartments [55], are less sensitive to the transient levels in the ER. Analyses of TLR4 ER-retention time, association with MD-2 and the effects on trafficking down the secretory pathway in primary MCs are needed.

The activation of TLR4 provides a pro-survival signal to MCs [56]. In addition, inclusion of the TLR4 inhibitor TAK242 suppressed the inflammatory response to mannan-induced histamine release [57]. In contrast, using TLR4 KO mice, it was shown that TLR4 increases systemic IL-10 levels and decreases surface expression of FcεRI on MCs, suggesting an LPS-mediated desensitization of MCs [58]. We found in CBMCs, less pronounced in PMCs (Figure 6), an additive effect of TLR4 and FcεRI stimulations in cytokine synthesis and secretion. The additive effect between TLR4 and FcεRI in human MCs was observed for IL-13 secretion [59], an important mediator of mucus production in allergic responses. Accordingly, the pharmacological inhibition of IRE1α in CBMCs diminished the additive effect of TLR4 and FcεRI in stimulation. The effect was smaller for PMCs, most likely due to the limited contribution of TLR4 stimulation to the overall effect and because FcεRI stimulation in murine MCs does not activate IRE1α. Our data suggest that IRE1α inhibition can be a strategy for disengaging the cooperation between TLR4 and FcεRI pathways and can provide a molecular explanation of why this synergism is more robust in human than in mouse MCs.

## 4. Materials and Methods

### 4.1. Chemical Reagents

Alcian Blue 8GX, safranin O, lipopolysaccharides (rough strains) from Salmonella enteric serotype Minnesota Re 595 (Re mutant), mouse IgE-anti-DNP, and JNK-IN-8 were all purchased from Sigma Aldrich (Saint Louis, MO, USA). Human myeloma IgE was obtained from Calbiochem-Merck (Darmstadt, Germany). Polyclonal rabbit anti-human IgE Ab was obtained from Dako (Copenhagen, Denmark). Goat anti-rat IgG F(ab)_2_ Ab was from Jackson ImmunoResearch Laboratories (Baltimore, MD, USA). DNP-BSA and Rat myeloma IgE were purchased from Invitrogen (Carlsbad, CA, USA). STF-083010 was synthesized by Tocris Bioscience (Bristol, UK). Thapsigargin was synthesized by Fermentek (Jerusalem, Israel), while DMSO was from Bio-Lab (Jerusalem, Israel).

### 4.2. Mice

MC-specific Mcpt5 promoter-driven Cre C57/BL6 mice were kindly provided by Dr. Axel Roers (Institute of Immunology, University Clinic Heidelberg, Heidelberg, Germany). IRE1α and XBP1 MC-specific knockout mice were generated by crossing Mcpt5-Cre to mice bearing floxed target alleles of XBP1 and IRE1α to homozygosity [29]. The mice were further crossed to the ROSA26 loxP-STOP-loxP YFP reporter mice, to report on the efficiency of the knockout by genetic labeling with YFP in the Cre-expressing cells. The generated three mouse lines were Mcpt5-Cre/YFP, Mcpt5-Cre/XBP1^fl/fl^/YFP (referred to as XBP1 KO), and Mcpt5-Cre/XBP1^fl/fl^/IRE1^fl/fl^/YFP (referred to as DKO). In all experiments, gender- and age-matched mice were used and housed under specific-pathogen-free conditions. 

### 4.3. Cell Culture

#### 4.3.1. Human CBMC Generation

Human MCs were obtained by culturing umbilical cord blood mononuclear cells as previously described [60]. In brief, fresh cord blood diluted in Hank’s solution was loaded on Ficoll-Hypaque (Amersham Pharmacia Biotech AB, Staffanstorp, Sweden) and centrifuged (350× *g*, 25 min, 26 °C). Mononuclear cells were washed twice and resuspended in a minimum essential medium (MEMα) supplemented with 10% heat-inactivated fetal bovine serum, 100 U/mL penicillin, 100 μg/mL streptomycin, and 10 μg/mL ribonucleases (all purchased from Biological Industries, Beit-HaEmek, Israel) and containing recombinant human stem cell factor (h-SCF; PeproTech, Cranbury, NJ, USA; 100 ng/mL) and human IL-6 (PeproTech, Cranbury, NJ, USA; 20 ng/mL). Prostaglandin E_2_ (PGE_2_; Sigma-Aldrich, Jerusalem, Israel; 1 ng/mL) was also added to this media regularly, in order to prevent any cultivation of monocytic contaminants. Cells were maintained in a humidified incubator (37 °C, 5% CO_2_) with media replaced on a weekly basis. Following a 7-week culture period, CBMCs were used following examination for maturity and viability. Cord blood was obtained according to the Institutional Helsinki Committee guidelines of Hadassah Hospital, and its use was approved by the committee.

#### 4.3.2. Murine BMMC and PMC Generation

BMMCs were generated from the femoral bone marrow of mice (six to eight weeks old) dissected in sterile conditions. Progenitors were maintained in an RPMI 1640 medium supplemented with 10% heat-inactivated fetal bovine serum, 100 U/mL penicillin, 100 µg/mL streptomycin, and 1× nonessential amino acids, all provided by Life Technologies Limited (Scotland, UK) and containing recombinant murine stem cell factor (m-SCF) and IL-3 (PeproTech, Cranbury, NJ, USA; 20 ng/mL each). Cells were maintained in a humidified incubator (37 °C, 5% CO_2_) with media replaced on a weekly basis until maturation at 4 weeks [61]. PMCs were obtained from mice peritoneal cavities by lavage with cold PBS. After abdominal massage (60 s), the cell suspension was collected again from the peritoneum, then centrifuged (160× *g*, 5 min, 26 °C), and the cell pellet was cultured for three weeks in a supplemented RPMI medium as mentioned before [62].

Prior to experimentation, cells were assessed for 90% viability by trypan blue exclusion, for 90% maturity by acidic toluidine blue staining, and for the expression of characteristic surface markers (KIT and FcεRI) by flow cytometry.

### 4.4. IgE-Mediated MC Activation

For activation of CBMCs, human myeloma IgE (0.3 μg/mL) was used for sensitizing the cells in the presence of recombinant human IL-4 (Peprotech, Cranbury, NJ, USA; 10 ng/mL,) for three days. Subsequently, cells were washed twice and resuspended in Tyrode’s buffer (consisting of 137 mM NaCl, 5.5 mM glucose, 2 mM KCl, 12 mM NaHCO_3_, and 0.3 mM Na_2_HPO_4_ and supplemented with 1.8 mM CaCl_2_ and 0.9 mM MgCl_2_; pH 7.34 for a short activation of 30 min) or in a CBMC medium supplemented with recombinant h-SCF (100 ng/mL) (for a longer activation). CBMCs were activated with polyclonal rabbit anti-human IgE Ab (5 μg/mL).

BMMCs and PMCs were sensitized overnight (18 h) with mouse IgE-anti-DNP (0.5 μg/mL) or with rat myeloma IgE (1 μg/mL). MCs were then washed and resuspended in Tyrode’s buffer (consisting of 135 mM NaCl, 5 mM KCl, 5.6 mM glucose, 20 mM HEPES, and 0.5 mg/mL BSA and supplemented with 1.8 mM CaCl_2_ and 1 mM MgCl_2_; pH 7.34) for 1 h of activation or in a supplemented RPMI medium (for longer activation). Cells (1 × 10^5^/100 μL) were activated with either DNP-BSA (10–100 ng/mL) or, as specified, goat anti-rat IgG F(ab)_2_ Ab (1 µg/mL) at 37 °C. Following activation, cells were centrifuged (160× *g*, 5 min, 4 °C), and supernatants and cell pellets were collected and kept frozen for an analysis of the mediators’ release.

### 4.5. β-Hexosaminidase Release Assessment

MC degranulation was evaluated by a chromogenic assay for β-hex. β-hex in the supernatants and cell lysate (lysed with 1% Triton X-100 in Tyrode’s buffer) was quantified by a hydrolysis of p-nitrophenyl-N-acetyl-β-D-glucopyranoside (Sigma Aldrich, Saint Louis, MO, USA) in a 0.1 M sodium citrate buffer (pH 4.5) for 120 min at 37 °C. OD was evaluated at a wavelength of 410 nm using a Cytation 3 plate reader (Agilent Technologies, Santa Clara, CA, USA). The percentage release of β-hex was calculated using the following formula:

Degranulation (%) = [OD supernatant/(OD lysate + OD supernatant)] × 100.

### 4.6. Flow Cytometry

For surface marker staining, cells (1 × 10^5^ cells per well) were seeded in a 96-well U-shaped plate and blocked with 5% goat serum for 15 min. Fluorophore-conjugated primary antibody was added according to the manufacturer’s instructions, followed by filtration through a 100 µM strainer directly to FACS tubes. The fluorophore conjugated antibodies used were APC-anti-mouse CD117 (c-kit) (eBioscience, San Diego, CA, USA, ab17-1171-81), PE-anti-mouse FcεRIα (BioLegend, San Diego, CA, USA, ab134308), APC anti-mouse/human CD282 (TLR2) (BioLegend, San Diego, CA, USA, ab153005), and PE/Cyanine7 anti-mouse TLR4 (CD284)/MD2 complex (BioLegend, San Diego, CA, USA, ab117609). PE Armenian Hamster IgG (eBioscience, San Diego, CA, USA, ab12-4888-83) and APC-Rat IgG2b kappa (eBioscience, San Diego, CA, USA, ab17-4031-81) were used as isotype controls.

Cells were acquired by BD FACSCalibur (BD Biosciences, Franklin Lakes, NJ, USA) or Cytoflex FACS (Beckman, Indianapolis, IN, USA) and analyzed using FlowJo v10 software (BD Life Sciences, Ashland, OR, USA) or CytExpert 2.3 software (Beckman Coulter, Indianapolis, IN, USA), respectively. Cells were gated according to physical parameters and to the specific staining used.

### 4.7. Cell Sorting

Peritoneal mast cells (PMCs) were sorted based on the YFP reporter to exclude the non-recombined PMCs. PMCs isolated from XBP1 KO and DKO mice were sorted after the 3rd week of culturing. Cells were resuspended in PBS containing 3% FCS, 200 U/mL penicillin, and 200 μg/mL streptomycin and were filtered through a 0.7 μm filter. YFP-positive cells were sorted on a BD FACSAria III cell sorter (BD Biosciences, Franklin Lakes, NJ, USA) using a 488 nm laser and collected in a supplemented RPMI medium containing 200 U/mL penicillin and 200 μg/mL streptomycin.

### 4.8. Western Blotting

Cells were harvested, centrifuged (160× *g*, 5 min, 4 °C), and washed twice in PBS. For cell lysis, RIPA buffer supplemented with protease and phosphatase inhibitors was added in a volume about four times the volume of the cell pellet and then vibrated for 30 min at 4 °C. Lysates were cleared by centrifugation (18,000× *g*, 15 min, 4 °C). A fivefold reduced Laemmli sample buffer was added and boiled for 5 min at 95 °C. The protein concentrations of the lysates were determined using the QPRO-BCA Kit Standard (Cyanagen Reagents for Molecular Biology, Bologna, Italy). Equal protein amounts were separated on SDS-PAGE gel and consequently electrotransferred onto polyvinylidene difluoride membranes (Merck, Ireland, UK) using the PowerPac Basic Power Supply (Bio-Rad, Jurong, Singapore). The membranes were blocked with 5% skim milk in a Tris-buffered saline/Tween 20 buffer (TBST buffer) for 1 h at room temperature and then incubated with the primary Abs overnight at 4 °C. After washing twice with the TBST buffer, the membranes were incubated with HRP-conjugated secondary goat anti-rabbit (Jackson Immunoresearch, Baltimore, PA, ab111-035-144) at room temperature for 1 h. Ag/Ab complexes were visualized using an ECL detection kit (Immobilon Crescendo Western HRP Substrate, Millipore, Burlington, MA, USA). p97 or β-actin was used as a loading control, and the band intensity was quantified using ImageJ software [63].

The following primary antibodies were used: anti-phospho-IRE1α rabbit mAb [EPR5253] (phospho S724) (ab124945), purchased from Abcam (Cambridge, UK), as well as anti-IRE1α (14C10) rabbit mAb (ab3294), phospho-JNK (Thr183/Tyr185) mAb mouse antibody (ab9255), and SAPK/JNK antibody (total-JNK) pAb rabbit (ab9252), and β-actin pAb rabbit (4967), purchased from Cell Signaling Technology (Danvers, MA, USA). TLR4 mAb mouse (sc-293072) was obtained from Santa Cruz Biotechnology (Dallas, TX, USA). Polyclonal rabbit anti-p97 was provided by Dr. Hidde Ploegh (Boston Children’s Hospital, USA). Blots were developed in Bio-Rad ChemiDoc™ XR and analyzed using Image Lab™ 5.1 software (Bio-Rad Laboratories, Carlsbad, CA, USA).

### 4.9. RNA Preparation and Quantitative RT-PCR

Total RNA was extracted using an RNeasy Mini Kit (QIAGEN, Germany) according to the manufacturer’s instructions. RNA (0.5–1 µg) was reverse-transcribed into cDNA using the iScript^™^ cDNA Synthesis Kit (Bio-Rad Laboratories, Carlsbad, CA, USA) according to the manufacturer’s instructions. Quantitative Real-Time PCR was carried out using iTaq™ universal SYBR^®^ Green Supermix (Bio-Rad Laboratories, Carlsbad, CA, USA) in the CFX Connect™ Real-Time PCR Detection System (Bio-Rad Laboratories, Carlsbad, CA, USA). The real mRNA levels of the target genes were assessed using DCt values by normalization to β-actin or GAPDH by Bio-Rad CFX Manager 3.1 software (Bio-Rad Laboratories, Carlsbad, CA, USA). Specific primers are listed in Table 1 and Table 2.

RT-PCR, using MyCycler Thermal Cycler 115V (BioRad, USA), was also performed to determine XBP1 deletion efficiency based on the ratio of the distinguished delta band (352 bp) to flox band (183 bp), using the 2XPhire Green Hot Start II PCR Master Mix (Thermo Fisher Scientific Baltics UAB, Vilnius, Lithuania) with INT1-S, 5′-CTTTGTGGTCGTAGGGTAGGAACC-3′, lox-S, 5′-ACTTGCACCAACACTTGCCATTTC-3′, and lox-A, 5′-CAAGGTGGTTCACTGCCTGTAATG-3′, as well as the detection of XBP1 mRNA splicing: forward: 5′-ACACGCTTGGGAATGGACAC -3′; reverse: 5′-CCATGGGAAGATGTTCTGGG-3′.

### 4.10. ELISA Assays for Cytokines

For cytokine level measurement, the medium suspended CBMCs or PMCs (concentration: 0.5–1 × 10^6^ cells/mL) were incubated with the indicated treatments in a humidified atmosphere with 5% CO_2_. After incubation, centrifugation (160× *g*, 5 min, 4 °C) was conducted to collect supernatants, followed by an assessment of IL-6 or TNF by ELISA kits according to their instructions. The used kits were ELISA MAX™ Deluxe Set Human IL-6, ELISA MAX™ Deluxe Set Mouse IL-6, and ELISA MAX™ Deluxe Set Mouse TNF, which were all purchased from Biolegend, San Diego, CA, USA.

### 4.11. Light Microscopic Imaging of Alcian Blue/Safranin-Stained MCs

1 × 10^5^/100 μL cells from the MCs were cyto-spun at 300× g for 3 min, air-dried for 10 min, and fixed for 30 min in methanol, 40% formaldehyde, and an acetic acid solution, mixed in a ratio of (85:10:5) by volume. Slides were incubated for 30 min with a solution of 0.5% alcian blue 8GX in 0.3% acetic acid (pH 2.4). Slides were then washed in PBS and incubated for 5 min with a solution of 0.3% safranin O in 0.125 N acetic acid (pH 1). Finally, they were washed with PBS, air-dried, and mounted with a mounting medium. Cell images were taken from random 40× fields with a Zeiss AxioCam ICc5 color camera mounted on a Zeiss Axio Scope A1 light microscope (Zeiss, Stuttgart, Germany) and of each slide.

### 4.12. Confocal Microscopic Imaging of the Endoplasmic Reticulum of MCs

For visualization of the ER in the IgE-activated MCs, CBMCs, and PMCs were sensitized, as described earlier, in IBIDI μ-Slide 8-Well dishes (IBIDI GmbH, Munich, Germany; 5 × 10^4^ cells/well), which were precoated with a Poly-L-Lysine solution (PLS) (Sigma-Aldrich, Saint Louis, MO, USA) to allow for cell adherence. On the activation day, CBMCs and PMCs were activated with polyclonal rabbit anti-human IgE Ab or mouse IgE-anti-DNP, respectively, for 4 h at 37 °C. ER-Tracker™ Green (BODIPY^®^ FL Glibenclamide, Cell Signaling, Danvers, MA, USA) in a concentration of 2 μM was added, and the samples were photographed immediately using a Confocal A1R microscope (Nikon, Tokyo, Japan) and a 60× oil-immersion objective. Relative fluorescent intensity (RFI) was measured using Fiji 1.53c software (U. S. National Institutes of Health, Bethesda, MD, USA) [64].

### 4.13. Transmission Electron Microscopic Imaging of PMCs

PMCs isolated from WT, XBP1 KO, and DKO mice were sorted from the peritoneal lavage based on YFP. Cells were collected and fixed in 2.5% glutaraldehyde and 2% paraformaldehyde in a 0.1 M cacodylate buffer (pH 7.4) for 4.5 h at room temperature, then rinsed four times for 10 min each time in a cacodylate buffer, post-fixed, and stained with 1% osmium tetroxide and 1.5% potassium ferricyanide in a 0.1 M cacodylate buffer for 1 h. Cells were then washed four times in a cacodylate buffer, followed by dehydration in increasing concentrations of ethanol consisting of 30, 50, 70, 80, 90, and 95% for 10 min at each step, followed by 100% anhydrous ethanol three times for 20 min each time and propylene oxide twice for 10 min each time. Following dehydration, the cells were infiltrated with increasing concentrations of agar 100 resin in propylene oxide, consisting of 25, 50, 75, and 100% resin for 16 h at each step. The cells were then embedded in fresh resin and allowed to polymerize in an oven at 60 °C for 48 h. Embedded cells in blocks were sectioned with a diamond knife on Leica Reichert Ultracut S microtome, and ultrathin sections (80 nm) were collected onto 200-mesh thin-bar copper grids. The sections on the grids were sequentially stained with uranyl acetate and lead citrate for 10 min each and viewed with a Tecnai 12 transmission electron microscope (TEM) at 120 kV (Phillips, Eindhoven, the Netherlands) equipped with a Phurona camera and RADIUS software (Emsis GmbH, Münster, Germany).

### 4.14. Statistical Analyses

The statistical analysis of the experimental data was performed using Prism 9.3.1 (GraphPad Software, San Diego, CA, USA). Data are presented as the mean ± SD. All comparisons between groups were carried out using a two-tailed unpaired Student’s t-test. Differences were considered significant at *p* < 0.05 and are labeled with an asterisk (*) on each graph.

## Figures and Tables

**Table 1:** List of human primer sequences for RT-qPCR.

Human Genes	Forward	Reverse
sXBP1	AACCAGGAGTTAAGACAGCGCTT	CTGCACCTGCTGCGGACT
ERdj4	GGT GTG CCA AAA TCG GCA TC	GCACTGTGTCCAAGTGTATCATA
IRE1α	AGCATGAGGTGTGGCATTAG	ACAGAGCAGCCTCTTCATTTC
IL-6	CCTGAACCTTCCAAAGATGGC	CACCAGGCAAGTCTCCTCATT
TNF	CTGCACTTTGGAGTGATCGG	TCAGCTTGAGGGTTTGCTAC
β-actin	GCGAGAAGATGACCCAGATC	CCAGTGGTACGGCCAGAGG

**Table 2:** List of murine primer sequences for RT-qPCR.

Murine Genes	Forward	Reverse
TLR1	GGGAAAAAGAAGACCCCGAA	GACACATCCAGAAGAAAACGGAA
TLR2	TGTACCCTCAATGGGCTCGG	GGATATGCAACCTCCGGATAGTG
TLR3	TCTGGGCTGAAGTGGACAAAT	ACCTCAGGCTTGGGAGATAGG
TLR4	AGTGGTTGCTGTTCTTATTCTGATTTG	ACCCATGAAATTGGCACTCA
TLR5	AGCCCCGTGTTGGTAATATCTC	TGGTAGTATTGAGGATCCAGGGA
TLR6	GAATTTGGCAACCTGACGAAG	TGCAGCTTAGATGCAAGTGAGC
TLR7	ATCCACAGGCTCACCCATACTT	GCTAGACTGTTTCCTTGAACATTTG
TLR8	GTGCCATCTTCCATAAAGCG	TGCAGTTGACGATGGTTGCATTC
TLR9	TGCAATTGGCTGTTCCTGAA	GGTGGTGGATACGGTTGGAG
ERdj4	CTCCACAGTCAGTTTTCGTCTT	GGCCTTTTTGATTTGTCGCTC
sXBP1	AAGAACACGCTTGGGAATGG	CTGCACCTGCTGCGGAC
IRE1α	GAGCAAGCTAACGCCTACTC	CACCATTGAGGGAGAGGCAT
IL-6	TCCAGTTGCCTTCTTGGGAC	GTGTAATTAAGCCTCTGACT
TNF	AGCACAGAAAGCATGATCCG	TGCCACAAGCAGGAATGAGA
GAPDH	AGGTCGGTGTGAACGGATTTG	TGTAGACCATGTAGTTGAGGTCA

RT-PCR, using MyCycler Thermal Cycler 115V (BioRad, USA), was also performed to determine XBP1 deletion efficiency based on the ratio of the distinguished delta band (352 bp) to flox band (183 bp), using the 2XPhire Green Hot Start II PCR Master Mix (Thermo Fisher Scientific Baltics UAB, Vilnius, Lithuania) with INT1-S, 5′-CTTTGTGGTCGTAGGGTAGGAACC-3′, lox-S, 5′-ACTTGCACCAACACTTGCCATTTC-3′, and lox-A, 5′-CAAGGTGGTTCACTGCCTGTAATG-3′, as well as the detection of XBP1 mRNA splicing: forward: 5′-ACACGCTTGGGAATGGACAC -3′; reverse: 5′-CCATGGGAAGATGTTCTGGG-3′.

## Data Availability

Not available.

## Supplementary Materials

Supplementary materials can be found at https://www.mdpi.com/article/10.3390/ijms231911826/s1.
