# Supplementary material for: Endoplasmic Reticulum Homeostasis Regulates TLR4 Expression and Signaling in Mast Cells
Source: Int J Mol Sci. 2022 Oct 5;23(19):11826. doi: 10.3390/ijms231911826 (PMC9569687; doi:10.3390/ijms231911826)
Supplement: Supplementary file 1 [file ijms-23-11826-s001.zip › ijms-1906872-supplementary.pdf]

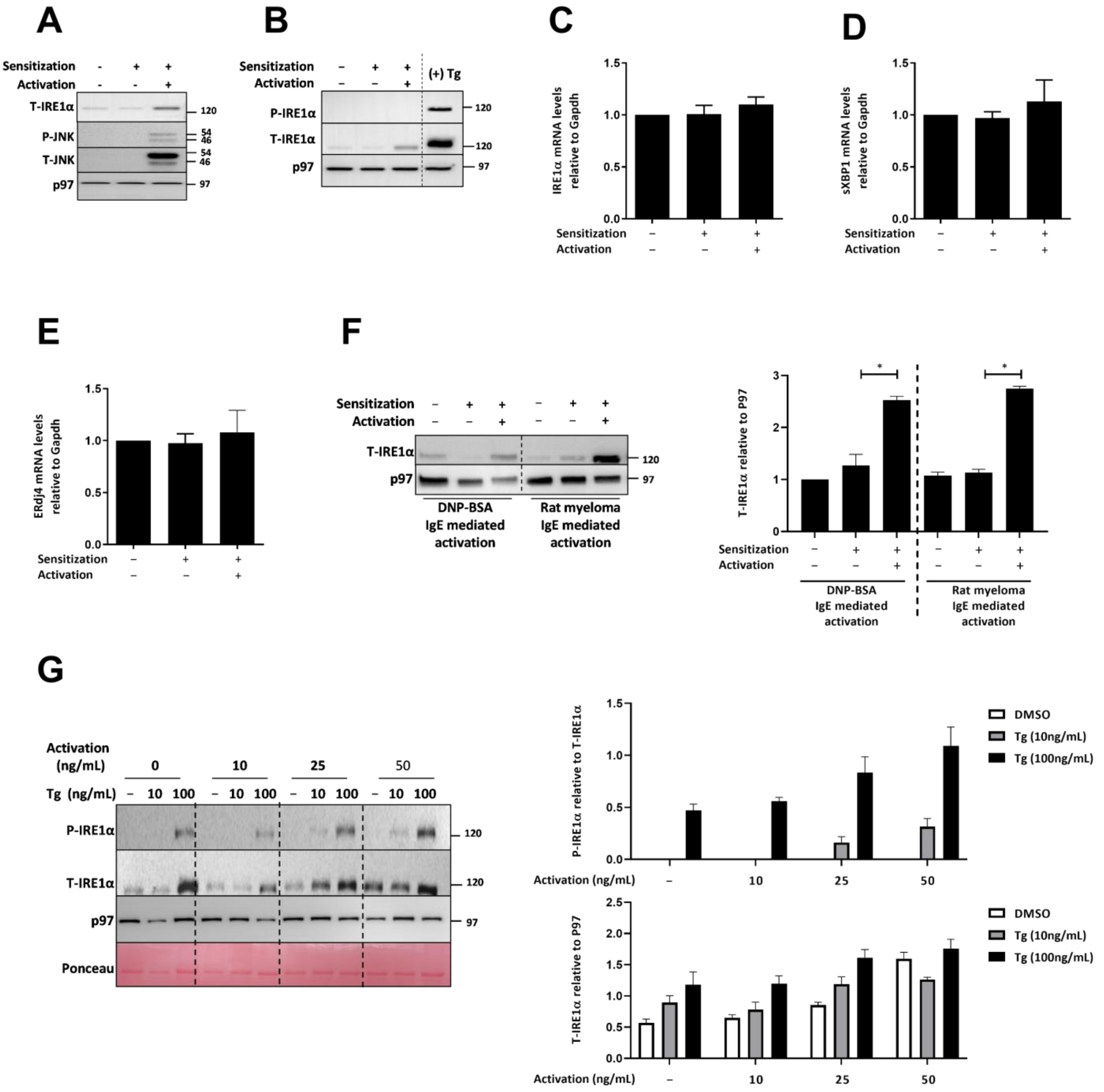


**Figure S1. ER expansion and upregulation in UPR transducers in BMMCs. (A)** WT BMMCs were sensitized with mouse IgE-anti-DNP (0.5 µg/ml, 18 h), followed by activation with DNP-BSA (50 ng/ml, 4 h). Equal whole cellular protein extracts were analyzed by immunoblotting for total IRE1α, phosphorylated and total JNK, and p97 as loading control. Shown is a representative image of three independent experiments. (B) WT BMMCs were treated for 4 h as mentioned in (A) or treated with Tg (1 µg/ml) as a positive control. Equal whole cellular protein extracts were analyzed by immunoblotting for phosphorylated and total IRE1α, and p97 as loading control. Shown is a representative image of three independent experiments. **(C)** RT-qPCR analysis of IRE1α,**(D)** sXBP1 and **(E)** ERdj4 following activation. Shown are the averages of relative mRNA levels of three independent experiments normalized to Gapdh ± SD. **(F)** BMMCs were sensitized overnight with mouse IgE–anti-DNP (0.5 µg/ml) or with rat myeloma IgE (1 µg/ml), followed by activation with either DNP-BSA (50 ng/ml) or with goat anti-rat IgG $F(ab')_2$ Ab (1 µg/ml), respectively, for 4 h. Total cellular protein extracts were analyzed by immunoblotting for total IRE1α and p97 as loading control and quantified by densitometry. **p*<0.05. **(G)** WT PMCs were sensitized using mouse IgE-anti-DNP (0.5 µg/ml, 18 h) and left either non-activated or activated with DNP-BSA (10, 25, and 50 ng/ml). 2 h later, cells were treated with DMSO or Tg (10 or 100 ng/ml) for additional 2 h. Total cellular protein extracts were analyzed by immunoblotting for phosphorylated and total IRE1α, and p97 as loading control, and quantified by densitometry. Shown are means ± SD of three independent experiments. **p*<0.05.

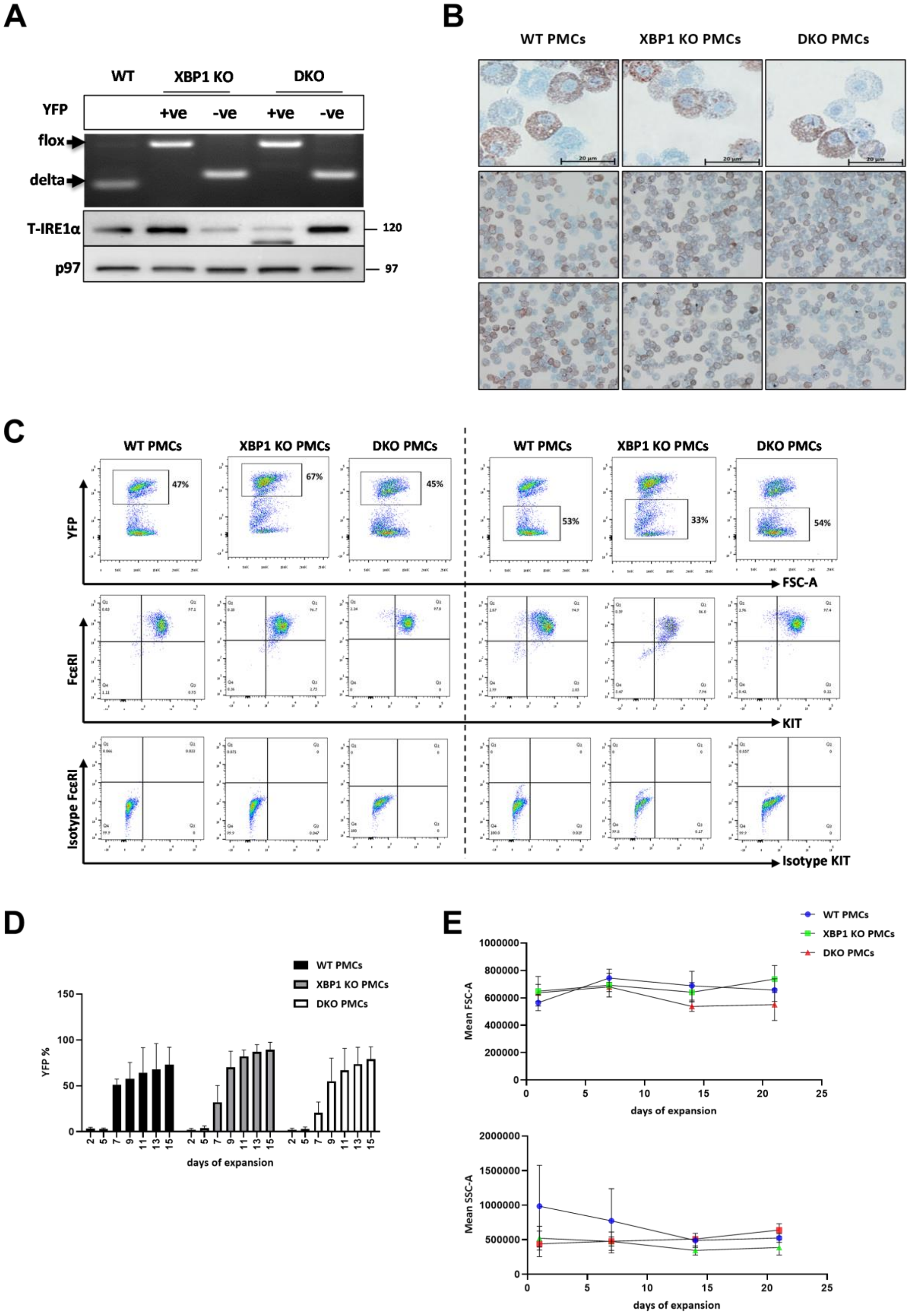


**Figure S2. The IRE1/XBP1 arm of the UPR does not affect the morphology, overall size and the proliferation/maturation of MCs. (A)** PMCs were generated from WT, MC-specific XBP1 KO, and MC-specific IRE1α/XBP1 DKO mice. YFP positive and negative mature MCs were separated by sorting and examined. PCR analyses of the delta and flox alleles were used to evaluate XBP1 deletion efficiency. **(B)** WT, XBP1 KO, and DKO PMCs were mounted on cytospin slides, stained with alcian blue and safranin, and analyzed by light microscopy at magnification of 40X. Shown are typical images. **(C)** FACS analysis of KIT and FcεRI expression on (left panel) YFP positive PMCs, (right panel) YFP negative PMCs. **(D)** Proliferation and maturation of PMCs were studied by monitoring YFP percentages in cell cultures by flow cytometry at 2, 5, 7, 9, 11, 13, and 15 days following the collection of the peritoneal lavage. **(E)** Peritoneal MC size was assessed by flow cytometry by tracking the mean FSC (upper panel) and SSC (lower panel) of YFP positive gated cells at 1,7,14 and 21 days following the collection of the peritoneal lavage. Data are shown as mean ± SD.

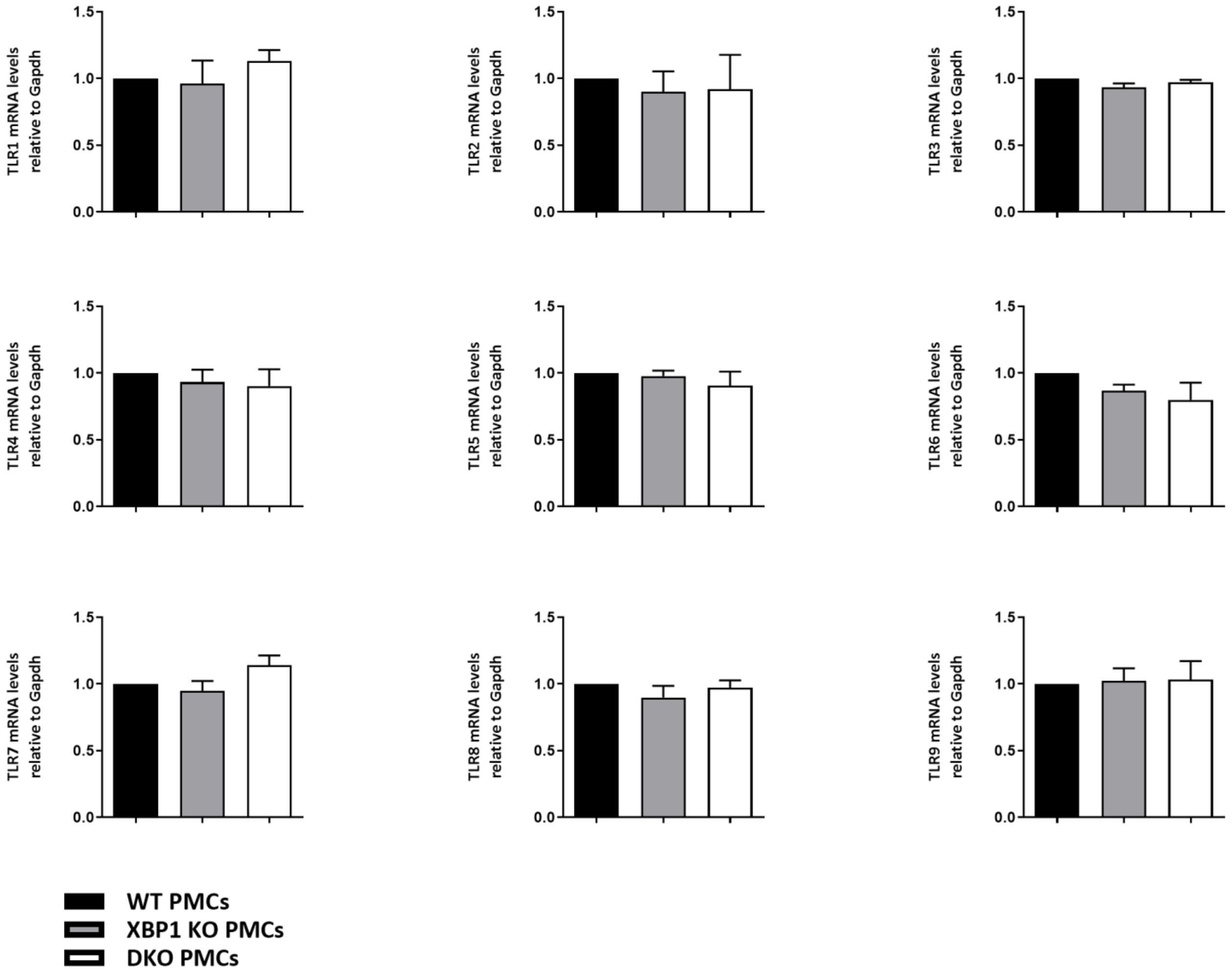


**Figure S3. TLR mRNA levels are not affected by IRE1/XBP1 pathway of the UPR**. RT-qPCR analysis of the gene levels of TLRs (TLR1-TLR9). Shown are the means of relative mRNA levels normalized to Gapdh ± SD of three independent cellular batches.
